# Changes in HbA1c Level over a 12-Week Follow-up in Patients with Type 2 Diabetes following a Medication Change

**DOI:** 10.1371/journal.pone.0092458

**Published:** 2014-03-25

**Authors:** Jennifer A. Hirst, Richard J. Stevens, Andrew J. Farmer

**Affiliations:** Department of Primary Care Health Sciences, University of Oxford, Oxford, United Kingdom and National Institute for Health Research School for Primary Care Research, Oxford, United Kingdom; University of Tolima, Colombia

## Abstract

**Background:**

Current guidance about the interval needed before retesting HbA1c when monitoring for glycaemic control is based on expert opinion rather than well-powered studies. The aim of our work was to explore how fast HbA1c changes after a change in glucose-lowering medication. This has implications for whether routine HbA1c testing intervals before 12 weeks could inform diabetes medication adjustments.

**Methods:**

This 12-week cohort study recruited patients from 18 general practices in the United Kingdom with non-insulin treated diabetes who were initiating or changing dose of oral glucose-lowering medication. HbA1c was measured at baseline and 2, 4, 8 and 12 weeks after recruitment. HbA1c levels at earlier time intervals were correlated with 12-week HbA1c. A ROC curve analysis was used to identify the 8-week threshold above which medication adjustment may be clinically appropriate.

**Results:**

Ninety-three patients were recruited to the study. Seventy-nine patients with no change in medication and full 12-week follow-up had the following baseline characteristics: mean±standard deviation age of 61.3±10.8 years, 34% were female and diabetes duration of 6.0±4.3 years. Mean HbA1c at baseline, 2, 4, 8 and 12 weeks was 8.7±1.5%, (72.0±16.8 mmol/mol) 8.6±1.6% (70.7±17.0 mmol/mol), 8.4±1.5% (68.7±15.9 mmol/mol), 8.2±1.4% (66.3±15.8 mmol/mol) and 8.1±1.4% (64.8±15.7 mmol/mol) respectively. At the end of the study 61% of patients had sub-optimal glycaemic control (HbA1c>7.5% or 59 mmol/mol). The 8-week change correlated significantly with the 12-week change in HbA1c and an HbA1c above 8.2% (66 mmol/mol) at 8 weeks correctly classified all 28 patients who had not achieved glycaemic control by 12 weeks.

**Conclusions/interpretation:**

This is the first study designed with sufficient power to examine short-term changes in HbA1c. The 12-week change in HbA1c can be predicted 8 weeks after a medication change. Many participants who had not achieved glycaemic control after 12 weeks may have benefitted from an earlier review of their HbA1c and medication.

## Introduction

Diabetes and its associated health complications are an increasing global health problem [1], [2]. Maintaining good glycaemic control is an essential part of routine diabetes care and a major contributor to minimising future complications [3]. The National Institute for Health and Clinical Excellence (NICE) in the United Kingdom (UK) currently recommend monitoring of glycated haemoglobin (HbA1c) every 2–6 months in people with type 2 diabetes [4]. Similarly the American Diabetes Association (ADA) guidelines [5] recommend performing the HbA1C test at least two times a year in patients who are meeting treatment goals (and who have stable glycemic control) and performing HbA1C tests quarterly in patients whose therapy has changed or who are not meeting glycemic goals [5].

Current clinical practice appears to be based on a belief that HbA1c tests cannot usefully be repeated within two or three months. [6] This is contingent upon the assumption that glucose binding with erythrocytes is irreversible [7]. However, some studies have demonstrated that it is likely that the glucose and haemoglobin interaction is actually a reversible process or that secondary reactions are taking place [8], [9]. Sacks and others in their 2011 guidelines [10] state that there is an absence of well-controlled studies to suggest a testing protocol and that there is *“There is no consensus on the optimal frequency of Hb A1c testing”.* Recommendations are therefore based on expert opinion and the only empirical evidence on short-term change in HbA1c comes from 2 small studies of 9 and 10 patients [11],[12]. Data from some studies has suggested that the rate at which HbA1c changes after a change in medication may be more rapid than previously thought, [13], [14] with clinically important changes in HbA1c, occurring within a period of 4–8 weeks [13], [15], [16].

Recent reports suggest that guidelines are not necessarily being followed; [14], [16], [17], [18], [19] for example in one UK trust 21% of all HbA1c tests were performed more frequently than guidelines recommend [17]. Over 80% of these requests came from primary care and over two thirds of the more frequent requests were for patients who had well-controlled diabetes. There is a need for an evidence base to inform clinical practice to avoid over use of tests without restricting circumstances where more frequent testing has a clinical benefit. We set out to explore the response of HbA1c to medication dose change before the conventional interval of twelve weeks and establish whether earlier measurement has potential for informing clinical management after a change in glucose lowering medication.

## Methods

We carried out a prospective primary care based cohort study in general practices in the Thames Valley region of the UK between July 2012 and May 2013. Ethical approval was obtained from the South-East Committee of the National Research Ethics Service. The study was registered on the UK National Institutes for Health Research Clinical Research Network Portfolio Database. We recruited patients with a diagnosis of type 2 diabetes of at least 3 months duration who were not taking insulin and were initiating or changing their type or dose of oral glucose lowering medication to lower their HbA1c as deemed necessary by their General Practitioner (GP). We excluded patients who were pregnant or breastfeeding, had a life-threatening illness or were unable to give informed consent. The duration of the study was 12 weeks.

All participants gave written informed consent prior to participation in the study. Data was collected on patient demographics, smoking status, diabetes medication type and dose before entering the study. The planned change in glucose lowering medication (dose and type of medication) was also recorded. Anthropometry included measurement of height, weight and waist circumference. At the baseline visit all participants had a venous blood sample taken for measurement of HbA1c and were asked to start taking their new glucose lowering medication as per usual care. Further venous blood samples were taken for HbA1c measurement at two, four, eight and twelve weeks after the baseline visit and medication change. HbA1c was measured in the venous blood samples in six central hospital laboratories (Stoke Mandeville, Royal Berkshire, John Radcliffe, Wexham Park, Milton Keynes and High Wycombe) using high performance liquid chromatography on National Glycohemoglobin Standardisation Program certified instruments [20] and following UK national accredited external quality control programmes (NEQAS or WEQAS). At each visit, medication adherence was recorded using the 8-item Morisky scale [21], and categorised using previously described methods [22] as high for patients with no positive answers; moderate adherence for patients with (on average) up to 2 positive answers out of 8; and low adherence for patients with (on average) more than 2 positive answers out of 8. Weight and waist circumference were re-measured at the final study visit as well as any changes in smoking status.

### Statistical Methods

The primary outcome measure was change in HbA1c from baseline 2, 4, 8 and 12 weeks after the medication change. Analysis was restricted to those with measurements at 0 weeks and 12 weeks and no intervening change in diabetes medication. Baseline characteristics of all eligible participants and the analysis population were reported.

We estimated that a sample size of 80 participants would allow us to measure a change in HbA1c of approximately 0.05% (assuming a SD of 0.1%) with 85% power. Statistical analyses were carried out in Stata 12 SE (StataCorp, Tx, USA). We calculated the mean change in HbA1c at 2, 4, 8 and 12 weeks relative to the baseline HbA1c value for each patient. We also reported change in weight and change in waist circumference during study participation. All results are reported as mean and standard deviation (SD).

Correlations of 2, 4 and 8 week change in HbA1c with 12 week change in HbA1c were calculated using Pearson’s correlation coefficients. Multiple linear regression was used to determine whether change in HbA1c at 2, 4 and 8 weeks could be used to predict HbA1c at 12 weeks. Each of the models was compared to a linear regression model of HbA1c at 12 weeks against HbA1c at 0 weeks. The p-value was calculated by the likelihood ratio method. These methods were repeated adjusting for differences in medication changes and medication adherence. We analysed these subgroups separately to examine differences in outcomes relative to medication adherence. Sensitivity analyses were carried out to exclude patients who did not have their 12-week HbA1c measurements within the protocol-specified time window (within 3 days either side of exactly 12-weeks from baseline visit).

In a *post-hoc* analysis we generated a receiver operator curve (ROC) of the sensitivity and specificity of 8 week HbA1c to predict glycaemic control at 12 weeks, defined as HbA1c of 7.5% (59 mmol/mol) or below. We used this to calculate the 8 week HbA1c threshold at which patients would remain uncontrolled at 12 weeks, and therefore might benefit from having their medication dose increased prior to the conventional 12-week interval.

## Results

**Figure 1:**
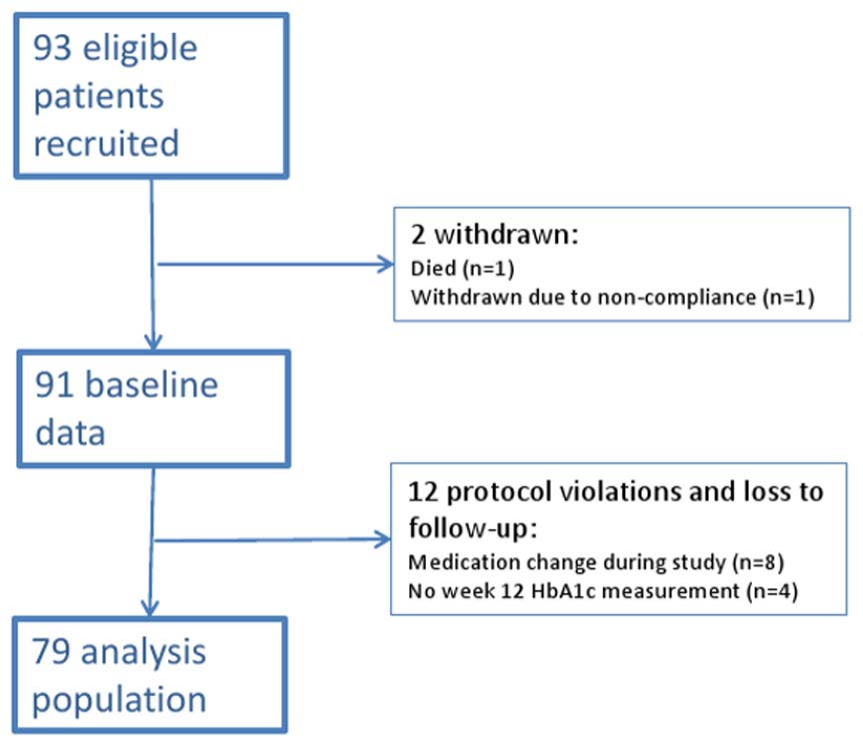
Study flow chart.

A total of 93 eligible patients were recruited to the study from eighteen health centres. Of those recruited, two patients were withdrawn from the study: one patient died and one failed to take their medication. Of the remaining 91, 8 patients changed their type or dose of medication before end of follow-up and an additional 4 patients did not attend for a 12-week HbA1c measurement leaving 79 patients for analysis. A flow chart of study recruitment and loss to follow-up is shown in Figure 1. The 79-patient analysis population had the following baseline characteristics (shown in Table 1): mean (sd) age was 61.3 (10.8) years, 27 (34%) were female, diabetes duration of 6.0 (4.3) years and baseline HbA1c of 8.7±1.5% (72.0±16.8 mmol/mol). The index medication change was a dose increase of an existing drug in 39 of 79 patients, new drug in 39 patients, and new drug plus dose increase of existing drug in 1 patient. Baseline characteristics for all recruited patients and the excluded patients were similar (Table 1).

**Table 1:** Baseline characteristics of included patients.

	All study participants	Analysis population	Excluded participants
	Mean (sd)	Mean (sd)	Mean (sd)
N	91	79	12
Age (years)	61.2 (10.4)	61.3 (10.8)	60.6 (8.0)
Female n (%)	30 (33.0%)	27 (34.2%)	3 (25%)
Duration of diabetes (years)	6.2 (4.6)	6.0 (4.3)	7.6 (6.9)
Baseline HbA1c (mmol/mol)	72.3 (16.8)	72.0 (16.8)	74.6 (17.3)
Smoking status:			
Current smoker	16%	15%	18%
Former smoker	53%	51%	64%
Never smoked	31%	33%	18%
BMI (kg/m^2^)	40.0 (9.0)	39.7 (8.5)	41.9 (11.7)
Waist circumference (cm)	109.5 (15.0)	109.7 (15.3)	108.0 (13.2)
Ethnicity	98.8% white	98.7% white	100% white
% taking antihypertensives	63%	61%	75%
% taking lipid lowering medication	76%	76%	75%
***Baseline medication:***			
diet controlled	13%	12%	18%
metformin	51%	52%	45%
sulfonylurea	4%	4%	
metformin+sulfonylurea	32%	32%	36%
***Index therapeutic change:***			
increase sulfonylurea	24%	24%	25%
increase metformin	22%	22%	25%
new sulfonylurea	22%	23%	17%
new metformin	13%	13%	17%
new sitagliptin	12%	11%	17%
new pioglitazone	2%	3%	
increased pioglitazone	2%	3%	
2 medication changes	2%	3%	

Note: Percentages are rounded to the nearest whole digit and may not add to 100%.

**Figure 2:**
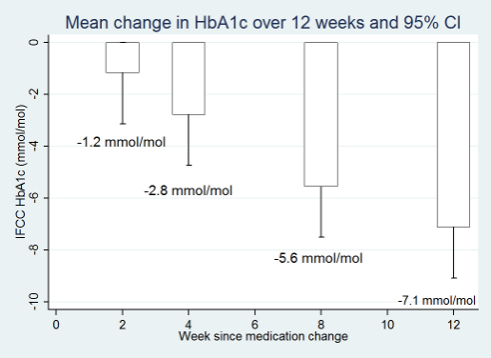
Mean change in HbA1c in mmol/mol at 2, 4, 8 and 12 weeks after an increase in diabetes medications and 95% confidence intervals.

**Figure 3:**
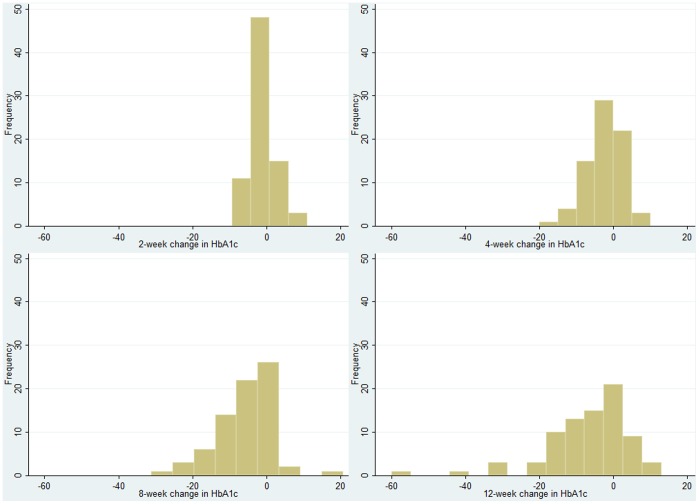
Histograms showing spread of change in HbA1c 2, 4, 8 and 12 weeks after a change in diabetes medication.

Mean±standard error (se) change in HbA1c, shown in Table 2 and Figure 2, was −0.11±0.04% (−1.2±0.4 mmol/mol) after 2 weeks, −0.25±0.05% (−2.8±0.6 mmol/mol after 4 weeks, −0.51±0.08% (−5.6±0.9 mmol/mol) after 8 weeks and −0.65±0.11% (−7.1±1.3 mmol/mol) after 12 weeks. Results were similar when two patients who had two simultaneous medication changes were excluded from the analysis. When thirteen patients who did not have their 12 week HbA1c measured within 3 days of 12-weeks from their baseline visit were excluded in a sensitivity analysis, results for the remaining 67 patients were similar (Table 2). These 67 patients had mean change (±se) in HbA1c at 2, 4, 8 and 12 weeks of −0.12±0.04% (−1.4±0.4 mmol/mol), −0.27±0.05% (−3.0±0.6 mmol/mol), −0.47±0.09% (−5.1±1.0 mmol/mol) and −0.56±0.11% (−6.1±1.2 mmol/mol) respectively. There was a small non-significant overall increase in mean (sd) weight (0.16±2.73 kg) and decrease in waist circumference (−0.75±5.0 cm) of participants during study participation. Histograms were plotted to show the distribution in the change in HbA1c at each measurement point (Figure 3); these show that there is an increase in the spread of the change in HbA1c at each measurement point and that some patients had an increase in their HbA1c after their baseline measurement and medication change.

**Table 2:** Mean (sd) HbA1c and change in HbA1c in mmol/mol for all patients and excluding patients with measurements outside 3-day window.

Week	All patients			Sensitivity analysis		
	Mean HbA1c	Change in HbA1c	n	Mean HbA1c	Change in HbA1c	n
0	72.0 (16.8)	–	79	70.9 (16.3)	–	67
2	70.7 (17.0)	−1.2 (3.4)	77	69.2 (16.1)	−1.4 (3.3)	66
4	68.7 (15.9)	−2.8 (4.8)	74	67.9 (15.6)	−3.0 (4.7)	65
8	66.3 (15.8)	−5.6 (7.8)	75	66.3 (15.7)	−5.1 (8.1)	64
12	64.8 (15.7)	−7.1 (11.1)	79	64.8 (15.4)	−6.1 (9.9)	67

By week 12, thirty-one of the 79 patient analysis population (39%) had achieved glycaemic control. Forty-eight patients who did not achieve control at 12 weeks had a much smaller overall mean change in their HbA1c (−0.3±0.7%, −3.6±7.8 mmol/mol) compared with those who did achieve control (−1.1±1.2%, −12.6±13.3 mmol/mol). Forty-nine patients (62%) had not yet achieved glycaemic control at 8 weeks; of these five did achieve control after 12 weeks though none had an HbA1c below 7.1% (54 mmol/mol).

Forty-two patients achieved a greater reduction in HbA1c at 12 weeks than at 8 weeks, but 37 had either the same or higher HbA1c levels at 12 weeks compared with their 8 week levels. Sixteen patients (20%) had higher HbA1c levels 12 weeks after their medication change than they had at baseline; fourteen of these patients had already exceeded their baseline HbA1c after 8 weeks.

**Figure 4:**
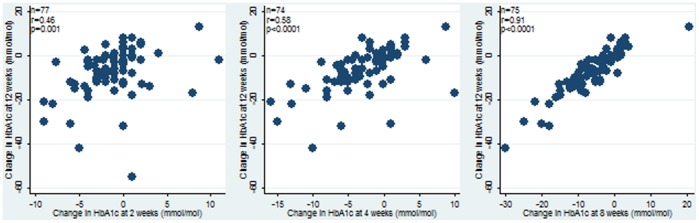
Correlation between 2 week, 4 week and 8 week HbA1c versus change in12 week HbA1c in mmol/mol.

Figure 4 shows graphs of changes in HbA1c at weeks 2, 4 and 8 plotted against change in HbA1c at 12 weeks. Each one of these showed a significant correlation between change at the earlier testing times and the 12-week change in HbA1c with corresponding correlation coefficients of 0.46 (95% CI 0.27 to 0.62), 0.58 (95% CI 0.41 to 0.72) and 0.91 (95% CI 0.87 to 0.95) respectively. Linear regression modelling showed a significant correlation between change in HbA1c at 2, 4 and 8 weeks with change in HbA1c at 12 weeks, with coefficients (SE) of 1.18 (0.35), 1.21 (0.20) and 1.16 (0.06) and R^2^ values of 0.13, 0.34 and 0.83 respectively. The change in HbA1c at each time interval was significantly predictive of the 12-week change in HbA1c (p = 0.001 at 2 weeks, p<0.0001 at 4 and 8 weeks). Among patients who had their 12-week HbA1c tested within 3 days either side of the date 12 weeks from baseline, the correlations of change in HbA1c at 2, 4 and 8 weeks with the 12-week change, with correlation coefficients were 0.52, 0.58 and 0.92 respectively, slightly higher than those for the full analysis population.

Table S1 shows change in HbA1c stratified by high, moderate and low medication adherence. The 15 most adherent patients had a mean change in HbA1c after 12 weeks of −0.5±1.2% (−5.6±13.1 mmol/mol), 50 patients with moderate medication adherence had a mean change in HbA1c of −0.7±1.1% (−7.3±11.7 mmol/mol) and 14 patients who had the lowest reported medication adherence had a mean 12-week change in HbA1c of −0.7±0.6% (−8.0±6.5 mmol/mol). Correlation of the 8 week change with 12-week change was highest in the most adherent patients (R^ = ^0.96).

Thirty-eight patients who received an increase in the dose of a single medication had a mean decrease in their HbA1c by 0.2±0.6% (2.2±6.6 mmol/mol) and 0.3±0.7% (2.7±7.4 mmol/mol) at 8 and 12 weeks respectively. Thirty-nine patients who initiated a new type of medication had a mean decrease in their HbA1c of 0.8±0.6% (8.4±6.8 mmol/mol) and 1.0±1.1% (10.7±11.6 mmol/mol) at 8 and 12 weeks respectively. A detailed breakdown of medication type and change in HbA1c is shown in Table S2.

The ROC curve analysis (Figure S1) showed that 8-week HbA1c correctly classified the majority of the 12 week HbA1c with an area under the curve of 0.954 (95% CI 0.912 to 0.996). For example eight-week HbA1c measurements of 8.2% (66.1 mmol/mol) or above had 100% sensitivity for predicting the people who were still be poorly controlled at 12 weeks; alternatively, of 42 patients who were above an HbA1c cut-off of 7.8% (62 mmol/mol) at 8 weeks, 39 (93%) remained uncontrolled at 12 weeks.

## Discussion

### Key Findings

Our study has provided evidence that earlier and more frequent measurement of HbA1c after a change in medication has potential to inform adjustments of medication in patients with diabetes. In addition, this is the first study on this scale designed and powered specifically to explore the course of HbA1c change over the weeks following a change in medication dose and suggest a minimum testing interval based on evidence from patient data. In this cohort of patients we found that 79% of the change in HbA1c had occurred within the first 8 weeks of a medication change and that this result remained robust in sensitivity analyses. The results of our study show that the HbA1c level 8 weeks after a change in medication was strongly predictive of HbA1c 12 weeks after the change in diabetes medication and that patients with HbA1c greater than 8.2% (66 mmol/mol) at 8 weeks did not achieve glycaemic control at 12 weeks.

### Compared to the Literature

Current guidance on the optimal testing frequency for HbA1c draws on extrapolation from physiological data and there is a lack of consensus on recommendations in guidelines with an absence of well-designed studies [10], [16]. A minimum repeat testing interval for HbA1c of 12 weeks is typically suggested [4], [5], [10], [23] citing consensus reports, expert opinion or two small studies (total sample size n = 19). These studies [11], [12] were designed to understand the kinetics of HbA1c change and predict new steady state HbA1c as opposed to suggesting optimal testing intervals. In our study we have prospectively collected, for the first time, patient data from which it would be possible to derive evidence to inform optimal repeat testing intervals in patients who have had a medication change. Our study uses a patient cohort, larger than previous studies and typical of patients in a primary care setting, and a design that allows us to determine the value of testing earlier than the oft-cited 12 weeks. We have shown that by 8 weeks after medication change it is possible to identify many patients who will remain uncontrolled at 12 weeks and for whom an earlier adjustment to their medication is likely to be beneficial. We have also been able to compare glycaemic control in patients with different levels of self-reported medication adherence and patients taking different medication types.

### Limitations

We only followed up patients for 12 weeks and our analysis has made the approximating assumption that a new steady state in HbA1c has been reached at this point. Our data supports this in that many of the patients had already achieved their maximum change by 8 weeks. Additional support comes from a previous study [12] which followed 9 patients for 16 weeks and found that 98% of the 16-week change in HbA1c had occurred within the first 12 weeks. We do, however recognise that some patients, particularly those who have not been adherent to their medication, or those taking some types of medication may continue to experience change in their HbA1c beyond 12 weeks from their medication change. Our results may therefore not generalise to patients taking such medications: specifically pioglitazone, which was taken by only two patients in our study. Pioglitazone has active metabolites which are thought to result in extended glucose-lowering effects [24] and therefore HbA1c may continue to change over a longer time compared to other glucose-lowering medications. Although we used a validated scale [21] to measure medication adherence, the data collected was self-reported and may not therefore accurately reflect the actual medication taken. The extent of missing data in our study is very small, and more than compensated for by the size of patient cohort which has enabled us to detect a 0.05% change in HbA1c with more than 80% power. Although this 6-centre study is potentially subject to inter-laboratory differences, this is minimized by the use of certified instruments and accredited external quality control schemes, and further mitigated by the use of change in HbA1c as the primary outcome.

### Clinical Implications

In our cohort of 79 patients with uncontrolled diabetes we have demonstrated that the majority of the change in HbA1c has taken place within the first 8 weeks of a medication change. We have shown from our analysis that twenty-eight patients who had an HbA1c at 8 weeks of greater than 8.2% (66 mmol/mol) would have safely benefitted from an adjustment in their medication 8 weeks after their medication change. In addition our work has shown that people with uncontrolled diabetes could have their HbA1c re-tested after 8 weeks to identify those who are non-adherent or non-responders to their medication type. We recognise that any medication changes would need to be assessed in a case-by-case basis and need to be dependent on individual patients, their HbA1c level and combinations of medications which they are taking. A randomised trial to test an 8-week testing interval compared with usual care in people with uncontrolled diabetes is now needed.

## Supporting Information

Figure S1 ROC curve for predictive value of 8 week value of control at 12 weeks.
(TIF)

Table S1 Mean (sd) change in HbA1c in mmol/mol by medication adherence.
(DOCX)

Table S2 Mean (sd) change in HbA1c in mmol/mol by medication adherence.
(DOCX)
