# Supplementary figures and images for: Changes in HbA1c Level over a 12-Week Follow-up in Patients with Type 2 Diabetes following a Medication Change
Source: PLoS One. 2014 Mar 25;9(3):e92458. doi: 10.1371/journal.pone.0092458 (PMC3965408; doi:10.1371/journal.pone.0092458)

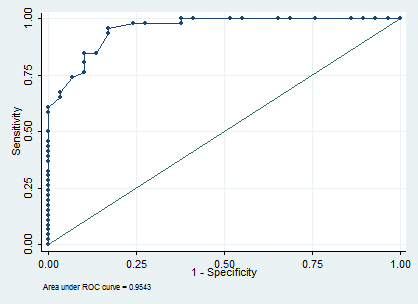

Supplement: Figure S1 — ROC curve for predictive value of 8 week value of control at 12 weeks. (TIF) [file pone.0092458.s001.tif]
