# Supplementary material for: Changes in HbA1c Level over a 12-Week Follow-up in Patients with Type 2 Diabetes following a Medication Change
Source: PLoS One. 2014 Mar 25;9(3):e92458. doi: 10.1371/journal.pone.0092458 (PMC3965408; doi:10.1371/journal.pone.0092458)
Supplement: Table S1 — Mean (sd) change in HbA1c in mmol/mol by medication adherence. (DOCX) [file pone.0092458.s002.docx]

**Table S1– Mean (sd) change in HbA1c in mmol/mol by medication adherence**

| **Week** | **High adherence (n=15)** | **Moderate adherence (n=50)** | **Low adherence (n=14)** |
| --- | --- | --- | --- |
| **Baseline** | **72.3 (19.6)** | **70.7 (14.1)** | **76.1 (22.6)** |
| 2 | -0.88 (3.8) | -1.1 (3.6) | -1.8 (2.3) |
| 4 | -1.6 (6.1) | -2.9 (4.4) | -3.6 (4.5) |
| 8 | -5.0 (9.7) | -5.5 (7.7) | -6.2 (6.0) |
| 12 | -5.6 (13.1) | -7.3 (11.7) | -8.0 (6.5) |
