# Supplementary material for: Changes in HbA1c Level over a 12-Week Follow-up in Patients with Type 2 Diabetes following a Medication Change
Source: PLoS One. 2014 Mar 25;9(3):e92458. doi: 10.1371/journal.pone.0092458 (PMC3965408; doi:10.1371/journal.pone.0092458)
Supplement: Table S2 — Mean (sd) change in HbA1c in mmol/mol by medication adherence. (DOCX) [file pone.0092458.s003.docx]

**Table S2-Mean change (sd) in HbA1c in mmol/mol by medication change**

| **Week** | **New metformin** | **New sulfonylurea** | **New sitagliptin** | **Increase metformin** | **Increase sulfonylurea** |
| --- | --- | --- | --- | --- | --- |
|  | n=10 | n=12 | n=9 | n=17 | n=19 |
| Baseline | 69.8 (13.8) | 74.8 (18.0) | 74.1 (18.3) | 68.3 (21.6) | 67.5 (9.3) |
| 2 | 0.3 (5.1) | -2.0 (4.2) | -2.7 (1.2) | -1.2 (2.0) | -0.38 (3.2) |
| 4 | -1.2 (5.0) | -5.3 (6.4) | -4.0 (2.5) | -2.5 (3.9) | -1.4 (4.7) |
| 8 | -4.9 (6.1) | -11.3 (6.8) | -5.0 (3.5) | -2.6 (5.2) | -1.1 (7.4) |
| 12 | -5.0 (8.0) | -15.6 (13.2) | -5.7 (5.1) | -3.4 (6.4) | -1.2 (7.7) |
| Achieved control | n=3 | n=11 | n=3 | n=9 | n=3 |

*2 patients who were taking pioglitazone and 2 patients who were given 2 medications together are not included in this table due to insufficient numbers.
